# Intraoperative use of fibrin glue in blepharoplasty: a possible solution to reduce postoperative complication

**DOI:** 10.1038/s41598-023-40183-9

**Published:** 2023-08-10

**Authors:** Kangmin Lee, Minwook Chang

**Affiliations:** 1 Department of Ophthalmology, Dongguk University Ilsan Hospital, 27, Dongguk-ro, Ilsandong-gu, Goyang-si, Gyeonggi-do, Republic of Korea; 2 Graduate School of Medical Science and Engineering, Korea Advanced Institute of Science and Technology (KAIST), Daejeon, Republic of Korea; 3 Shiley Eye Clinic, Hwa Sung, Republic of Korea

**Keywords:** Medical research, Cardiovascular diseases, Eye diseases, Haematological diseases

## Abstract

The purpose of this study was to investigate the effects of intraoperative fibrin glue use on surgery for blepharoptosis. This retrospective study included patients with acquired blepharoptosis who underwent surgical correction and were followed for at least one month. Patients were classified into two groups depending on whether treated with antithrombotic agents or otherwise. All patients taking antithrombotic agents discontinued with the treatment one week prior to surgery in accordance with our clinical guidelines. Preoperative and postoperative marginal reflex distance 1(MRD1) and ecchymosis grade were evaluated and compared. The subjects were 56 patients (111 eyes) who discontinued antithrombotic agents before surgery and 59 patients (117 eyes) who had never taken antithrombotic agents. Fibrin glue was used in 13 patients (26 eyes, 23.4%) in the antithrombotic group, and 11 patients (21 eyes, 17.9%) in the non-antithrombotic group. The rate of severe ecchymosis was significantly lower in patients who used fibrin glue in the antithrombotic group at 1 week (11.5 vs 40.0%, p = 0.008). However, in non-antithrombotic group, there was no difference in the rate of severe ecchymosis according to the use of fibrin glue at 1 week (14.3 vs 30.2%, p = 0.181). In patients with a history of taking antithrombotic agents, the intraoperative use of fibrin glue is thought to be helpful as it could significantly reduce significant ecchymosis.

## Introduction

Blepharoptosis is defined as an abnormal clinical condition characterized by low-lying upper eyelid margin in the primary gaze, which may affect both the function and appearance of the eyes^1,2^. The main purpose of treatment for blepharoptosis is that patients may benefit from both cosmetic and functional improvement with increased field of vision and quality of life^3^. Although the main treatment for blepharoptosis is surgical correction, it is difficult in that the both functional and cosmetic aspects need to considered, and there is a limit to predict the postoperative outcome. The complications that can occur after eyelid surgery include intra- or postoperative bleeding, infection, damage of lacrimal gland, and asymmetric eyelid crease^3,4^. Most of surgeons have been interested in major bleeding events such as retrobulbar hemorrhage, compartment syndrome, and orbital apex syndrome, and previous studies have been focused on these severe complications^5,6^. However, postoperative ecchymosis and edema, which are common conditions after surgery, are causing serious concern in that they make it more difficult to predict the surgical outcome by complicating to evaluate whether potential over- or under-correction after surgery^7,8^. Therefore, oculoplastic surgeons try to avoid such unwanted complications as much as possible, but studies investigating postoperative ecchymosis and edema are insufficient. The postoperative ecchymosis and edema could be associated with intraoperative or postoperative bleeding and there are several factors that could affect the bleeding associated with surgery, one of which most important is the use of antithrombotic agents^9,10^.

Antithrombotic agents, which include antiplatelet and anticoagulant, are some of the most commonly prescribed drug along with increased life expectancy and interest in cardiovascular disease^11,12^. It is widely reported that preoperative antithrombotic treatment increases the risk of intraoperative and postoperative bleeding^13–15^. In addition, the importance of bleeding events have been emphasized in the field of ophthalmic plastic and reconstructive surgery, the interest in the risk of bleeding due to the use of antithrombotic agents is rising. In common, oculoplastic surgeons recommend discontinuing the use of antithrombotic agents based on cardiologist consultation before surgery. However, our previous study reported that significant bruising and ecchymosis may occur after surgery for blepharoptosis even if discontinuing antithrombotic agents before surgery^16^. These results presented the importance of careful approaching to surgery in patients with history of taking antithrombotic agents.

Fibrin glue (FG) is composed of biomaterials that participate in coagulation (fibrinogen, thrombin, transglutaminase, calcium, etc.) and is generally used in areas where it is difficult to use sutures or in situations requiring rapid adhesion and hemostasis. FG was approved by the FDA in 1998 and started to be used on the human body with commercialized products such as Tisseel^®^ (Baxter, Vienna, Austria), Greenplast^®^ (Green Cross, Yongin, Korea), Beriplast^®^ (Aventis Behring, Marburg, Germany), Hemaseel^®^ (Haemacure, Sarasota, Floida, USA). It has been mainly used for skin amputation wounds or general surgical operation, but its usefulness has recently been reported that use of FG is expanding to the ophthalmologic field. However, little is known about the clinical efficacy of the FG in eyelid surgery. In addition, discontinuing the use of antithrombotic agents, which has been considered as one of the most important methods for reducing intra- and post-operative bleeding, could be insufficient in patients with history of taking antithrombotic agents. Therefore, our clinical study investigated the surgical effect of intraoperative fibrin glue use in patients with blepharoptosis, especially those with a history of taking antithrombotic agents.

## Results

During the study period, a total 152 patients were diagnosed with blepharoptosis and underwent surgical correction. Finally, of 115 patients eyes were included in this study with excluding 4 patients under 18 years of age, 5 patients with a history of eyelid surgery, 5 patients with follow-up less than one month, 8 patients with inaccurate measurement of MRD1 because of low quality of photographs, 6 patients who failed to discontinue antithrombotic agents, and 9 patients with hematological abnormalities found in laboratory tests. There were 56 patients who discontinued antithrombotic agents before surgery (antithrombotic group) and 59 patients who had never taken antithrombotic agents (non-antithrombotic group).

In antithrombotic group, intraoperative FG was used in 13 patients (23.4%). The mean age was 70.1 ± 11.7 years for those who used FG and 69.5 ± 8.1 years for those who did not use FG (p = 0.762). The mean duration of treatment in antithrombotic used group was 82 months, ranging from 11 to 120 months, showing no difference according to the use of FG. In addition, there was no significant difference in the proportion of males according to the use of FG (53.8% vs 34.9%, p = 0.332). The prevalence of hypertension (69.2% in FG used patients, 60.5% in FG not used patients, p = 0.747) and diabetes mellitus (30.8% in FG used patients, 44.2% in FG not used patients, p = 0.525) showed no difference. Forty-eight patients (85.75%) were treated with one agent and 8 patients (14.25%) were treated with dual-therapy. The rate of ‘severe ecchymosis’ in the antithrombotic group was significantly lower in patients who used FG 1 week after surgery (11.5% vs 40.4%, p = 0.008). There were no cases of ‘severe ecchymosis’ at 1 month after surgery in all patients of antithrombotic group. However, the rate of ‘persistent ecchymosis’ was significantly lower in patients using FG (11.5% vs 32.9%, p = 0.045) (Table 1).

**Table 1:** Baseline characteristics of antithrombotic group.

Parameters	Fibrin glue used (N = 13) Mean (SD)	Fibrin glue not used (N = 43) Mean (SD)	*p* values*
Age	70.1 (11.7)	69.5 (8.1)	0.762
Sex, male, N (%)	53.8	34.9	0.332
Hypertension, N (%)	69.2	60.5	0.747
Diabetes mellitus, N (%)	30.8	44.2	0.525
Severe ecchymosis one week after surgery, N (%)	11.5	40.4	0.008
Persistent ecchymosis one month after surgery, N (%)	11.5	32.9	0.045
Preoperative MRD1, mm	1.32 (0.65)	1.32 (0.61)	0.948
Postoperative MRD1, mm	3.11 (0.34)	3.06 (0.29)	0.646

*MRD1* marginal reflex distance 1.

*p* value by *t* test and Chi-square test (*p* < 0.05).

In non-antithrombotic group, intraoperative FG was used in 11 patients (17.9%). The mean age was 74.5 ± 8.4 years for those who used FG and 68.9 ± 8.6 years for those who did not use FG (p = 0.008). There was no significant difference in the proportion of males according to the use of FG (63.6% vs 33.3%, p = 0.089). The prevalence of hypertension (27.3% in FG used patients, 47.9% in FG not used patients, p = 0.316) and diabetes mellitus (18.2% in FG used patients, 29.2% in FG not used patients, p = 0.710) showed no difference. There was no significant difference in the rate of ‘severe ecchymosis’ following the use of FG 1 week after surgery in non-antithrombotic group (14.3% vs 30.2%, p = 0.181). Also, there was no case of ‘severe ecchymosis’ at 1 month after surgery in all patients, and there was no significant difference in the rate of ‘persistent ecchymosis’ according to the use of FG (9.5% vs 8.3%, p > 0.999) (Table 2).

**Table 2:** Baseline characteristics of non-antithrombotic group.

Parameters	Fibrin glue used (N = 11) Mean (SD)	Fibrin glue not used (N = 48) Mean (SD)	*p* values*
Age	74.5 (8.4)	68.9 (8.6)	0.008
Sex, male, N (%)	63.6	33.3	0.089
Hypertension, N (%)	27.3	47.9	0.316
Diabetes mellitus, N (%)	18.2	29.2	0.710
Severe ecchymosis, N (%)	14.3	30.2	0.181
Persistent ecchymosis, N (%)	9.5	8.3	> 0.999
Preoperative MRD1, mm	1.40 (0.79)	1.33 (0.74)	0.708
Postoperative MRD1, mm	3.04 (0.43)	2.99 (0.35)	0.856

*MRD1* marginal reflex distance 1.

*p* value by *t* test and Chi-square test. (*p* < 0.05).

There was no difference in MRD1 between the antithrombotic group and the non-antithrombotic group according to the use of FG at 1 week and 1 month after surgery. The degree of improvement of eyelid height evaluated by the difference in MRD1 before and 1 month after surgery did not show a significant association with the use of FG in both groups.

## Discussion

Fibrin glue (FG) for adhering living tissue was first used in cardiovascular surgery by Spangler in 1976^17^. Since then, it has been commercialized with FDA approval in the United States in 1998 and used throughout general surgical operations, but studies on the use of FG in oculoplastic surgery, including eyelid surgery, are lacking. Mandel reported that the use of autologous tissue before skin sutures was effective for wound closure and significant hemostasis in eyelid surgery^18^. In addition, Mommaerts reported that the use of commercialized FG in lower eyelid blepharoplasty had a significant effect on the occurrence of postoperative scarring^19^. However, although the use of FG has been used to reduce intraoperative and postoperative bleeding in other surgical field, there has been no report on the clinical effect of FG in reducing intraoperative or postoperative bleeding for eyelid surgery.

Oculoplastic surgeons struggle with whether or not to stop taking antithrombotic drugs before eyelid surgery. Although it is recommended to discontinue taking antithrombotic drugs before surgery in many previous studies, little is known about the effectiveness of discontinuing antithrombotic drugs in intraoperative or postoperative outcomes. Therefore, there has been controversy among reports due to the necessity and risks of discontinuing antithrombotic drugs. As few existing studies investigated the risk of taking antithrombotic agents and the need to discontinue them in ophthalmic surgery, in fact, most studies focused on cataract surgery or retinal surgery, so there was a limit to their application to oculoplastic surgery^20,21^.

Recent studies have reported that the use of antithrombotic agents has no significant effect or clinical problems on oculoplastic surgery. Philip et al. argued that the incidence of major bleeding in oculoplastic surgery was as low as 0.4% and there was no significant difference depending on whether or not antithrombotic agents were taken^22^. In addition, Bartely reported that antithrombotic agents did not cause significant bleeding complications in oculoplastic surgery, including eyelid surgery and dacryocystorhinostomy^23^. In fact, the American College of Chest Physicians (ACCP) has classified oculoplastic surgery as a low-risk group with a rate of 0–2% of severe bleeding occurrence after surgery, recommending not to stop taking antithrombotic agents^24^.

However, studies have also been reported of the risks of taking antithrombotic agents in oculoplastic surgery. Esparaz et al. argued that most oculoplastic surgeons experienced serious bleeding complications either during or after surgery in patients with taking antithrombotic agents^25^. In addition, Parkin et al. showed the higher rate of serious complications, such as retrobulbar hemorrhage, occurred in patients who had eyelid surgery with a history of taking antithrombotic agents^26^. Also, it is thought that the effectiveness of antithrombotic agents may be underestimated in that studies so far have mainly focused on serious complications rather than complications that can be easily encountered in clinical practice. In fact, in author’s previous study, we showed that there were significantly more bleeding events such as bruise or ecchymosis in patients who discontinued antithrombotic agents than the control groups^16^.

In current study, the history of taking antithrombotic agents and the use of FG were investigated in patients diagnosed with blepharoptosis and underwent surgical correction. The degree of ecchymosis, MRD1, and the improvement of eyelid height at 1 week and 1 month after surgery was compared according to the use of FG. There was no significant difference in MRD1 after surgery related to the use of FG in both groups. In the antithrombotic group, the rate of ‘severe ecchymosis’ 1 week after surgery and the rate of ‘persistent ecchymosis’ at 1 month after surgery was also significantly lower in the patients using FG. In contrast, the use of FG did not show a significant effect in non-antithrombotic group. The possible mechanism was that the application of FG could exhibit an effective hemostatic effect by blocking the hole of the blood vessel and preventing bleeding with the sealant and adhesive properties as previously reported^19^.

The mechanism of how FG aids in blood clotting is through the interaction of two main components: fibrinogen and thrombin. These two components interact with each other, converting fibrinogen in the plasma into fibrin monomers, which then polymerize to form a fibrin clot, inducing blood clotting at the site of application. Therefore, for patients who are taking antithrombotic agents, the effect of FG could be more pronounced. This is because antithrombotic agents inhibit blood clotting, and in such cases, the ability to promote blood clotting becomes even more crucial. On the other hand, for patients who are not taking antithrombotic agents, their blood clotting mechanisms are not significantly compromised, and the additional effect of FG on blood clotting may not be as prominent. Therefore, in surgeries with minimal bleeding or in cases where blood clotting is not a significant concern, the difference in effectiveness of FG may not be as noticeable.

Complications such as bruising or ecchymosis after eyelid surgery can be easily considered not serious complications such as retrobulbar hemorrhage, but it is of great clinical importance in terms of quality of life, such as return to daily life according to the cosmetic aspect and recovery speed. Therefore, clinicians should reduce possible bruising or ecchymosis after surgery in patients who have taken antithrombotic agents, so our studies tried to investigate the efficacy of using FG in patients who have taken antithrombotic agents and found that there was a significant complication-reducing effect.

Nevertheless, this study has some limitations. First, it is a retrospective study with a relatively small sample size, which may limit the statistical power. Second, there is a limitation in that we cannot quantify the amount of bleeding during surgery, which can be an accurate objective indicator. From the reason, the grade of ecchymosis used in the author’s previous study was applied, and it was intended to be supplemented by substituting whether it persisted or not at postoperative 1 month. Also, the grading of ecchymosis and persistence can be proxy and subjective. To reduce this subjectivity, we tried to adopt a case in which four masked graders evaluated equally. Lastly, this study did not consider the difference between performing surgeries on both eyelids simultaneously and operating on a single eyelid. When two surgeries were performed simultaneously, it was expected that the relatively longer duration of the combined procedure might contribute to more severe bruising. Therefore, further evaluation and interpretation are needed to investigate this aspect.

In conclusion, this study showed that the use of FG reduced the severity of postoperative ecchymosis in patients with a history of taking antithrombotic agents. It is a relatively simple possible solution that can reduce complications that may occur after eyelid surgery, so it is expected to help patients recover quickly.

## Methods

### Patients and data collection

This is a retrospective study of patients with acquired blepharoptosis who underwent surgical correction at the Department of Ophthalmology of Dongguk University Ilsan Hospital from January 2016 to January 2020. The study was approved by the Institutional Review Board of Dongguk University, Ilsan Hospital, Goyang, South Korea (IRB no. 2021-04-024-002), and the all data collections and researches were conducted following the tenets of the Declaration of Helsinki. The informed consent required was waived because of the retrospective nature and the minimal risk of this study. From our database, we obtained a list of patients who were diagnosed with blepharoptosis and underwent surgical correction, and retrospectively reviewed the patient’s medical records. All patients underwent ophthalmologic evaluation, including slit-lamp biomicroscopy, measurement of marginal reflex distance1 (MRD1) before and after surgery, and laboratory evaluation to exclude abnormal hematological findings. Patients treated with antithrombotic agents were included only if their discontinuation was recommended by a cardiologist, and educated to restart taking antithrombotic agents 48 h after surgery. Additional data including age, sex, duration of treatment, hypertension, diabetes mellitus, type of antithrombotic agents, the number of antithrombotic agents used were obtained. The exclusion criteria were as followed: (1) age < 18 years; (2) a history of previous eyelid or eyebrow surgery; (3) no follow-up for more than 1 month; (3) inaccurate measurement of marginal reflex distance1 (MRD1) before and after surgery; (4) systemic or neurodegenerative disease that may affect eyelid function; (5) hematologic disorder or abnormality laboratory results revealed in preoperative evaluation; (6) patients who failed to discontinue with antithrombotic agents due to the risk of thromboembolic events, or who did not voluntarily discontinue.

### Surgical technique and intraoperative fibrin glue use

**Figure 1:**
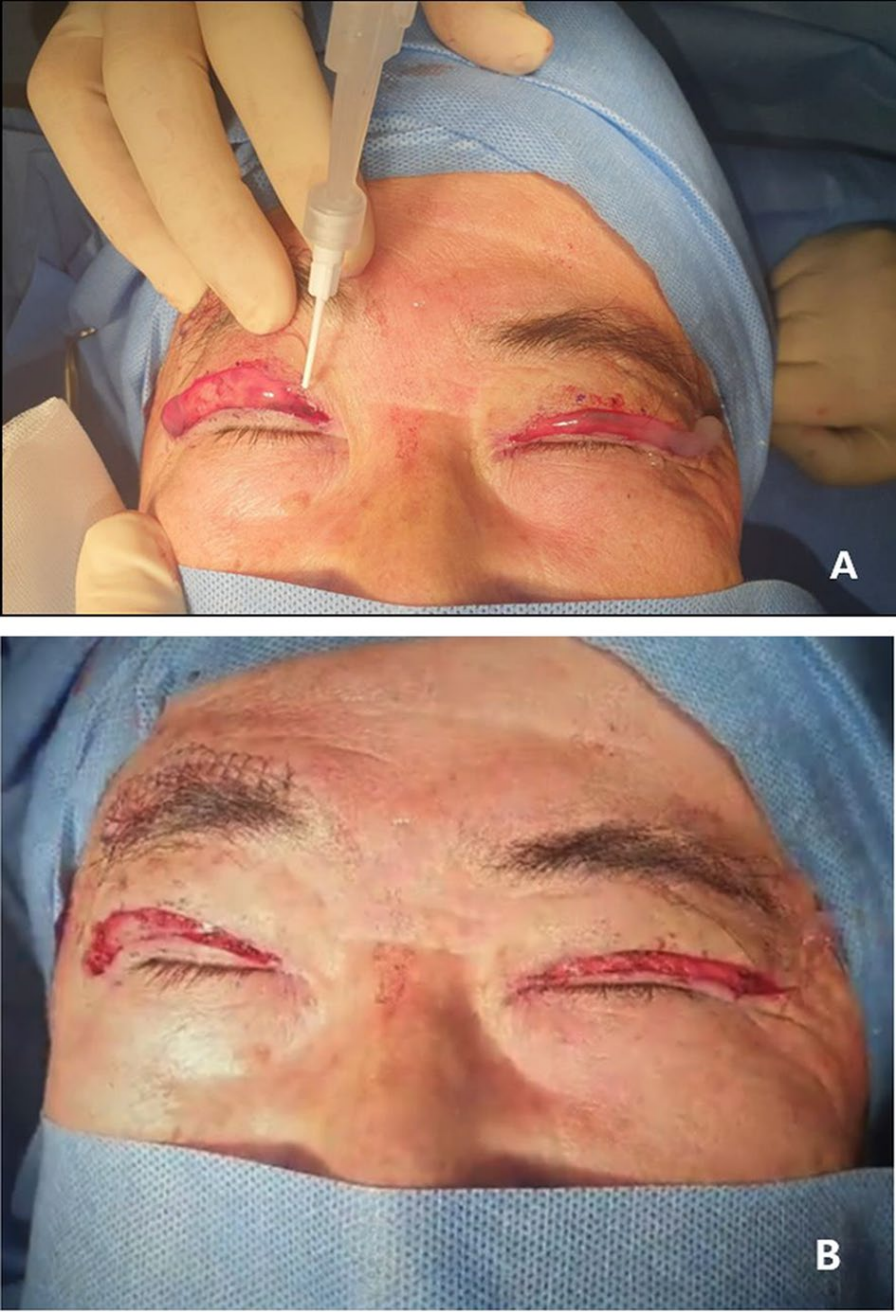
Photographs of a patient who underwent surgery for blepharoptosis. (**A**) Intraoperative photograph shows a procedure of applying fibrin glue to the surgical site before skin suturing. (**B**) Photograph after removing fibrin glue shows that rapid and effective hemostatic effect without significant additional bleeding.

Surgical correction of blepharoptosis was performed by one surgeon (M.C.). A skin incision was made under local anesthesia, and the dehiscence of the levator palpebral tendon was confirmed. And then, the levator aponeurosis and Müller's muscle were gently dissected from underneath conjunctiva with the thermal cautery and was fixed and sutured to the eyelid while confirming the release of levator aponeurosis. Before skin suturing, FG was applied topically to the surgical site and wiped out with sterile gauze after a few seconds (Fig. 1).

### Evaluation of MRD1 and ecchymosis grade

Preoperative and postoperative MRD1 at one week and one month were calculated using FIJI software (an expanded version of ImageJ version 1.51a, available at fiji.sc, free of charge). For measuring MRD1, the patients’ picture were taken with the primary gaze with fixed frontalis muscle and without any extra pressure, and MRD1 was automatically measured based on stickers with a dimeter of 8 mm attached to the patients’ forehead. It was manually measured five times by two masked examiner, and after excluding the highest and lowest values, the remaining three values were averaged.

**Figure 2:**
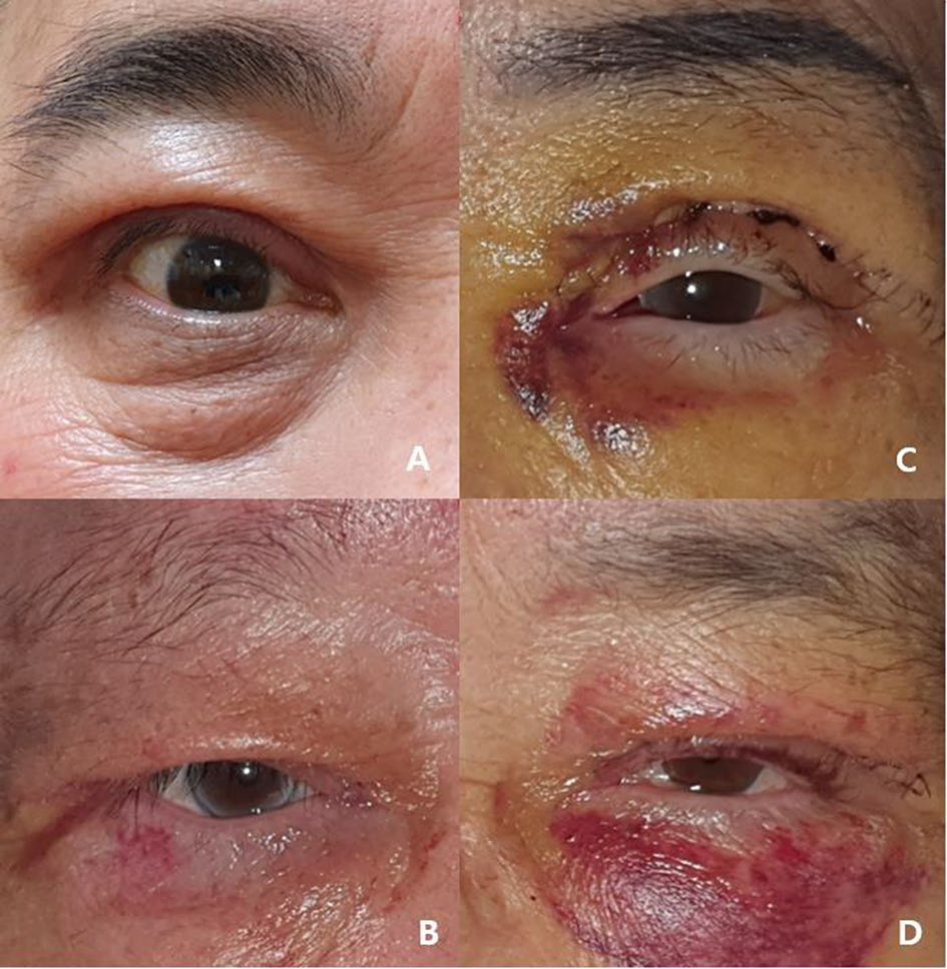
Photographs of example of postoperative ecchymosis grade. (**A**,**B**) Grade 0 and 1: no bruise or mild bruise (less than 1/2 of periorbital area). It is defined as ‘mild ecchymosis group’. (C)(D) Grade 2 and 3: moderate to severe bruise with dark purple color (more than 1/2 of periorbital area). It is defined as ‘severe ecchymosis group’.

The degree of postoperative ecchymosis was assessed with frontal facial view photographs according to previous reported ecchymosis grading system (Fig. 2). Ecchymosis grades were defined as follows: grade 0 = no visible ecchymosis, grade 1 = ecchymosis less than 1/2 of periorbital area, grade 2 = ecchymosis more than 1/2 of periorbital area with dark purple color), and grade 3 = ecchymosis beyond periorbital area. For statistical analysis, we classified grade 0 and 1 as ‘mild ecchymosis, and grades 2 and 3 as ‘severe ecchymosis’. To evaluate the degree of ecchymosis 1 month after surgery, ‘persistent ecchymosis’ was defined by the presence of ecchymosis 1 month after surgery regardless of grade. The rate of ‘severe ecchymosis’ and ‘persistent ecchymosis’ after surgery were compared and analyzed according to whether preoperative antithrombotic agents used and whether intraoperative FG was used.

### Statistical analyses

All statistical analysis was performed using IBM SPSS version 21.0 for Windows (SPSS Inc., Chicago. IL, USA). Independent two-sample *t* tests were used to compare normally distributed variables and Chi-square tests were used to analyze categorical variables. A value of p < 0.05 was considered as statistically significant difference.
